# SARS-CoV-2 and displaced persons in Afghanistan: A blind spot in epidemic preparedness

**DOI:** 10.7189/jogh.10.020308

**Published:** 2020-12

**Authors:** Benjamin CR Flower, Katherine MacDonald, Joanna Dobbin

**Affiliations:** 1 UN-Habitat, Kabul, Afghanistan; 2 Infectious Diseases, Royal Free Hospital, London, UK; 3 University College London, Department of Primary Care & Population Health, London, UK

Displaced populations living in overcrowded settlements present an emerging and severe COVID-19 public health risk in conflict-affected countries across the globe. In Afghanistan, the scale of the risk is sobering: over 8 million people have been displaced since 2012, including 4.2 million internally displaced people (IDPs) and 4 million returnees from Iran and Pakistan [1]. Many live in overcrowded urban settlements that lack basic water, sanitation and hygiene (WASH) services, where the virus and associated lockdowns can wreak human, social and economic havoc. In addition, forty years of protracted conflict have left the country with a fragile health infrastructure – there are less than 2000 isolation beds nationwide – that may exacerbate the human toll of the disease [2]. The spread of the virus is of immediate concern because of the country’s porous border with global hotspot Iran: since January 223 157 Afghans have returned, many fleeing the COVID-19 outbreak and settling in crowded urban settlements, presenting severe transmission risks [3]. In this context, this article assesses COVID-19 risk in urban IDP and returnee settlements by examining a displaced community in the eastern Afghan city of Jalalabad. In doing so, it provides insights into the causes of vulnerability and potential mitigating measures.

To date, there have been few empirical investigations of COVID-19 vulnerability in urban IDP and returnee settlements. However informal settlements around the world are becoming increasingly important sites in the fight against the pandemic. A recent case in Mumbai’s Dharvi – the world’s largest informal settlement – drew a swift containment response from Indian authorities [4]. Preventing transmission in these vulnerable settlements is crucial in reducing city-wide transmission, and an additional challenge to already stretched humanitarian and government entities in conflict zones.

The case of the settlement of Surkh Dewall in the Afghan city of Jalalabad is useful to highlight the public health and socioeconomic aspects COVID-19. The settlement consists of 643 households, who were displaced from the place of origin because of conflict between ISIS-K and government forces; 638 households were surveyed in 2019 as part of a UN-Habitat vulnerability assessment [5]. The case of Surkh Dewall demonstrates the many challenges associated with stemming the spread of the disease among displaced urban populations, and the socioeconomic risks associated with COVID-19 for the most vulnerable.

Like many informal settlements around the world, overcrowding and multiple occupancy housing presents a high risk of COVID-19 transmission for the population of Surkh Dewall. On average, there are over 15 people per dwelling, with the majority of households (92 per cent) residing in single roomed mud-brick dwellings. There is also a highly vulnerable households residing in tents, accounting for a total of 30 families and over 150 people. The high number of people living in close proximity is alarming because it has been known since January 2020 that the main spread of COVID-19 has occurred within households [6], driven by close contact and prolonged exposure to infected respiratory droplets. The ability for people to adhere to social distancing and to self-isolate, and thus protect others in their household when exhibiting symptoms of COVID-19, is near impossible in such a context.

Overcrowding synergises with low capacity WASH services to present additional transmission risks for residents. Limited access to running water, open defecation, and the price of hygiene items all impact on inhabitants’ ability to protect themselves from the spread of SARS-CoV-2. In this context, 9 per cent of households practice open defecation, with female-headed and lower-income households over represented in this bracket. The remainder of households use only basic open latrines, which also present high contamination risks, particularly in the context of overcrowded housing. These practices can lead to physical and psychological stress, and result in adverse health impacts that could complicate COVID-19 and result in worse health outcomes.

The precarious livelihood characteristics of residents of Surkh Dewall render them vulnerable to COVID-19 transmission, and the secondary socioeconomic impacts of the disease. Many of the residents are living in extreme poverty with just under a third having a family income of less than 1000AFG a month (US$13), equating to around US$ 0.08 per person per day; 50% of female-headed households were in this bracket. Residents reported informal sector livelihood activities, with the majority unskilled labourers (around 80 per cent), and the remainder as street vendors: such activities are at-risk of disruption due to government lockdowns, which are currently being implemented across Afghan cities. In this context, low and insecure incomes constrain residents’ ability to take measures to prevent COVID-19 transmission, such as purchasing personal hygiene products or staying at home and self-isolating when COVID-19 symptomatic. Households also have limited resources to meet the costs associated with health care and lost income if a family member becomes ill.

In the context of reduced income, food security has been highlighted by the World Food Programme as a key source of COVID-19 associated vulnerability [7]. In Surkh Dewall, a third of residents report food shortages, with women-headed households being most vulnerable. COVID-19 will likely exacerbate food insecurity. Disruption to supply chains has resulted in increased prices of staple foods across Afghanistan: wheat flour has increased by 15%-18% in price in local markets, cooking oil by 17% and rice and flour by 2%-4% [8]. These increases will push some of the most vulnerable families into severe food shortages; low and irregular income already resulted in pervasive food insecurity before the outbreak began.

**Figure:**
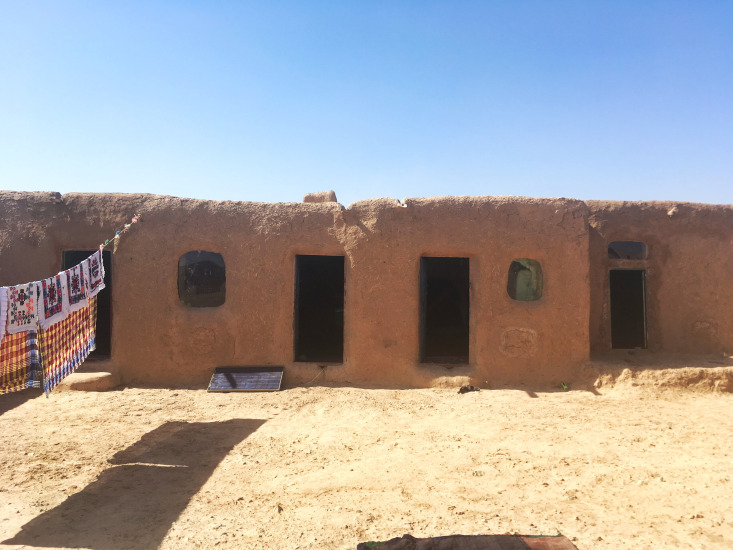
Photo: Mudbrick housing in IDP settlement in Afghanistan (copyright Naïk Lashermes 2020, used with permission).

A recent *Lancet* call to action stressed the need for vulnerable displaced populations to be included in the health sector’s COVID-19 response [9]. Building on this, the evidence from Afghanistan suggests that integrated responses need to address overcrowding and low-capacity WASH facilities, strengthen socioeconomic resilience and food security, and ensure gender sensitive and durable solutions to the crisis. Failing to strengthen the resilience of IDPs across Afghanistan will further damage public health outcomes across the country, and the region. Moreover, the findings demonstrate that protecting refugee and migrant health is paramount to the global SARS-CoV-2 response, and must be funded accordingly. The pandemic presents an opportunity for policy makers to recognise that good refugee and migrant health is good public health.
